# Waves and fluid–solid interaction in stented blood vessels

**DOI:** 10.1098/rspa.2017.0670

**Published:** 2018-01-17

**Authors:** S. Frecentese, L. P. Argani, A. B. Movchan, N. V. Movchan, G. Carta, M. L. Wall

**Affiliations:** 1 Department of Mathematical Sciences, University of Liverpool, Peach Street, Liverpool L69 7ZL, UK; 2 Department of Maritime and Mechanical Engineering, Liverpool John Moores University, 3 Byrom Street, Liverpool L3 3AF, UK; 3 Russells Hall Hospital, The Dudley Group NHS Foundation Trust, Pensnett Road, Dudley DY1 2HQ, UK

**Keywords:** wave propagation, quasi-periodicity, stented artery, dynamic fluid–structure interaction

## Abstract

This paper focuses on the modelling of fluid–structure interaction and wave propagation problems in a stented artery. Reflection of waves in blood vessels is well documented in the literature, but it has always been linked to a strong variation in geometry, such as the branching of vessels. The aim of this work is to detect the possibility of wave reflection in a stented artery due to the repetitive pattern of the stents. The investigation of wave propagation and possible blockages under time-harmonic conditions is complemented with numerical simulations in the transient regime.

## 1. Introduction

Cardiovascular disease (CVD) is the most common cause of mortality in adults within the Western world. The pathological process underpinning CVD is atherosclerosis, which can lead to narrowing and/or occlusion of blood vessels. The resulting reduction in blood flow velocity and volume causes tissue ischaemia (lack of oxygen delivery) in the territory supplied by the affected artery. Intraluminal stenting is one technique that can be performed to restore adequate flow and avoid ischaemia occurring. The outcomes of stenting vary depending on the anatomical site of the diseased arteries. For example, coronary stents have very good success rates in improving the patency of vessels, preventing further cardiac ischaemia and avoiding the need for surgical bypass. However, stenting of larger limb vessels in peripheral vascular disease has not been as successful and large amounts of energy and resources have been put into establishing why. The reason for this variability in stenting outcome is poorly understood and is likely to be multifactorial. Arterial walls are elastic and subjected to pulse waves originating from the left ventricle, the frequency and regularity of which are altered with physical activity and multiple disease states. It is a possibility that the reinforcement of arteries with stents alters the propagation of pulse waves through the arterial network altering the flow dynamics. This may lead to decreased flow velocity or increased wall shear stresses in the arterial wall that could induce changes leading to restenosis of occlusion.

Reflection of waves in blood vessels is a well-known phenomenon, but it has always been related to strong geometrical changes within the arterial tree, such as the branching of vessels [1,2]. The aim of this work is to investigate whether reflection of waves can occur in a stented artery, due to the reinforcement provided by the stents.

Wave propagation in fluid-filled cylinders has been extensively investigated in the literature. This problem, which involves fluid–structure interaction, has always been challenging: on the one hand, it is difficult to find solutions in a closed form, so that approximations and/or numerical techniques were employed; on the other hand, the study of the dynamics of fluid-filled cylinders constitutes the basis for the analysis of every piping system, which implies that it is suitable for a very broad range of applications. In particular, several investigations were conducted to determine the resonant frequencies and wave propagation in such systems, with [3–10] and without [11–27] the fluid.

Many computational models were performed for haemodynamics, but only a limited number of investigations addressed the propagation of waves in blood vessels [28] and, in particular, when the systems exhibit a repetitive pattern in their geometry [29]. A recent work by Jaganathan *et al*. [30] shows a comparison between different types of stents on the basis of their natural frequencies, but the analysis is performed only for the metallic structure.

The geometry of most of the commercially available stents is based on a brand-specific pattern consisting of the repetition of a *primitive cell* along the circumference of the structure, thus yielding the *unit cell* of the system (see [31] for stent geometry details). The deployed stent structure consists of the repetition of several unit cells along the axis of the vessel. These features suggest that a stented artery can be considered as a *periodic structure*, defined by a unit cell composed of the artery wall, the stent structure and the blood; hence, the system can be modelled as a fluid-filled periodically reinforced cylinder.

The analysis presented in the paper addresses three important points:

— the Bloch–Floquet waves in a periodically stented artery;
— the frequency response linked to the transmission/reflection problem for the case of a stented region of finite length; and
— the transient regime for a finite-length stented artery with nonlinear viscous fluid.

**Figure 1.**
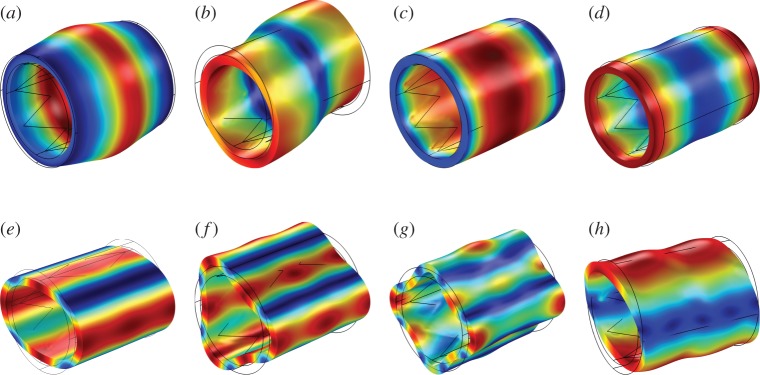
Representative shapes of the deformation modes observed in the unit cell with a stent. (*a*) Axisymmetric mode, (*b*) flexural mode, (*c*) torsional mode, (*d*) axial mode, (*e*) mode involving simple flattening of the wall, (*f*) mode involving trefoil flattening of the wall, (*g*) mode involving quatrefoiled flattening of the wall and (*h*) flexural-torsional mode with rotated end sections. (Online version in colour.)

The Bloch–Floquet approach is a useful technique to analyse the behaviour of periodic systems under harmonic perturbations. It is based on the study of a unit cell, which provides information on the dynamic properties of a periodic system, hence reducing the complexity of the problem. In particular, it allows to treat the stented artery as a waveguide and to identify the frequency ranges in which waves do and do not propagate. The application of this technique to fluid-filled periodically reinforced cylinders constitutes a novel approach for the detection of reflected waves in arteries in the absence of branching or any other sudden geometrical variation. The Bloch–Floquet analysis is also used to obtain the deformation modes of the stented blood vessel in the time-harmonic pulsation regime, as shown in figure 1.

Different types of stent configurations are investigated. In addition, two different cases are studied: one represented by a single stent, and the other in which the system consists of a cluster of stents separated by unstented sections. In the second case, standing waves are detected, which represent trapped modes in the unstented regions. A semi-analytical model is used to estimate the frequencies of these standing waves, which may fall within an interval of frequencies that can be experienced during daily activities.

The effects of propagation and attenuation of waves in terms of localization of strain and reduction of flow velocity are investigated by means of the frequency response analysis. This analysis confirms the results of the Bloch–Floquet approach and provides additional information on the physical properties of the system, such as pressure in the fluid and stress and strain in the arterial wall. The investigation is performed for an assembly of a finite number of unit cells subjected to an external harmonic loading. Moreover, different configurations are considered.

Finally, a transient regime analysis is performed for the finite-length stented artery, which shows in detail how the flow is affected by the reinforcements. In the transient computations, the fluid is described by the complete Navier–Stokes equations and full fluid–structure interaction is taken into account. This is a more realistic analysis and, compared with the above-mentioned techniques, provides more information on the system, such as the time history of the fluid velocity.

The text is organized as follows. Bloch–Floquet waves analysis is described in §2, and §3 is focussed on the case of a cluster of stents. In particular, §3b discusses a semi-analytical model for the evaluation of frequencies of trapped waveforms within the cluster of stents. Section 4 presents the transmission/reflection problem for the case of a stented region of finite length. Transient regime analysis is described in §5. Finally, general discussion and conclusions are presented in §6.

## 2. Waves in a periodically reinforced vessel

### (a) Governing equations

The Bloch–Floquet approach is used to analyse the waves that can propagate through the system and to determine their dynamic properties. This method allows one to derive the relation between the frequency and the wavenumber (or, equivalently, the Bloch–Floquet parameter). This relation is called the *dispersion relation* and its real solutions yield the so-called *dispersion curves*. The dispersion curves provide the group velocity (corresponding to the slope of the curve) and the phase velocity (secant slope) at each frequency. They also indicate the frequency ranges in which waves can physically propagate within the system (called *pass-bands*) and the ranges in which waves cannot propagate (called *stop-bands*). The Bloch–Floquet analysis reduces the problem to the study of a single unit cell which, in this case, includes the artery wall, the stent structure and the blood.

In the following, subscripts ‘a’, ‘s’ and ‘f’ in the equations denote the artery, the stent and the fluid (blood), respectively. Small displacement theory is employed in this work.

In order to determine the dispersion relation, the artery is modelled as a hollow cylinder composed of a linear elastic isotropic homogeneous material. Accordingly, its equations of motion are

2.1 $$\mu_{a}\nabla^{2}{\text{u}}_{a}+(\lambda_{a}+\mu_{a})\nabla (\nabla \cdot {\text{u}}_{a})=\rho_{a}\frac{\partial^{2}{\text{u}}_{a}}{\partial t^{2}},$$

where *μ*_a_ and *λ*_a_ are the Lamé parameters, *ρ*_a_ is the density (mass per unit volume), ***u***_a_ is the displacement vector, *t* is the time and ∇=(∂/∂*x*,∂/∂*y*,∂/∂*z*)^T^ represents the vector differential operator.

The blood is modelled as an acoustic medium, and its equation of motion is

2.2 $$K_{f}\nabla^{2}p_{f}=\rho_{f}\frac{\partial^{2}p_{f}}{\partial t^{2}},$$

where *p*_f_ and *K*_f_ are the pressure and the bulk modulus of the fluid, respectively, whereas *ρ*_f_ is the density of the fluid. This approximation yields accurate results within the framework of eigenfrequency analysis, as previously shown in the literature [32].

As the fluid is modelled as an acoustic medium, the coupling at the fluid–solid interface is obtained by means of the following relation for the stresses

2.3 $$\sigma_{a}\text{n}=-p_{f}\text{n},$$

where ***σ***_a_ is the stress tensor in the artery wall and ***n*** is the unit outward normal vector. The exterior boundary of the artery wall is free and this is expressed by the relation

2.4 $$\sigma_{a}\text{n}=\text{0}.$$

Analogous approaches describing interaction between an elastic medium and different sources in the time-harmonic regime have been proposed in the literature, see for instance [33].

It can be noted that the simplified time-harmonic computations are accompanied further by the full transient analysis of the fluid–structure interaction in the presence of the viscous Newtonian fluid, as discussed in §5. The interesting wave regimes identified in the linearized time-harmonic model are given additional attention in the transient computations.

In the linearized time-harmonic computations the stent is modelled as a curved wire with circular cross section composed of a linear elastic isotropic homogeneous material. The stents are considered to be already deployed and in contact with the artery wall. For simplicity, in this work the connection between the stent and the artery wall is assumed to be bilateral, which means that the stents are tied to the inner artery wall. Hence, continuity of displacements and tractions is assumed at the interface. No other constraints are applied in the model in order to allow for a broad class of deformation of the vessel. In fact, arteries themselves can be mobile with the movement of the body, including elongation and twisting [34,35].

#### (i) Bloch–Floquet waves

Time-harmonic regime is assumed. Hence, the displacement field in the artery, the pressure field in the blood and the displacement field in the stent are expressed as

2.5*a* $$\begin{array}{r} {\text{u}}_{a}(\text{x},t)={\text{U}}_{a}(\text{x})\;e^{i\omega t}, \end{array}$$

2.5*b* $$\begin{array}{r} p_{f}(\text{x},t)=P_{f}(\text{x})\;e^{i\omega t} \end{array}$$

2.5*c* $$\begin{array}{r} \;\mathrm{and}\;{\text{u}}_{s}(\text{x},t)={\text{U}}_{s}(\text{x})\;e^{i\omega t}, \end{array}$$

where ***U***_a_ and ***U***_s_ denote, respectively, the displacement amplitude vectors for the artery wall and for the stent, *P*_f_ is the pressure amplitude, and *ω* is the radian frequency.

**Figure 2.**
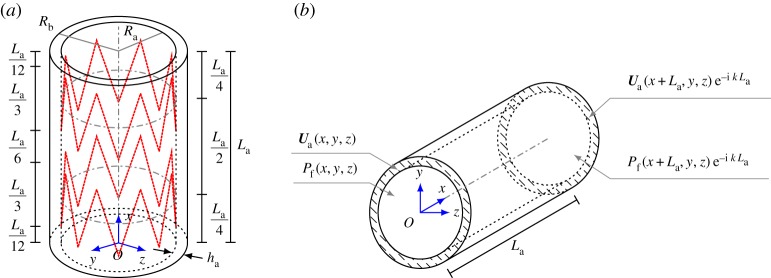
Representation of the geometry and of the quasi-periodic boundary conditions of the unit cell employed in the numerical simulations. The middle lines of the coils are represented by dotted lines in (*a*). The dashed-dotted circumferences shown in (*a*) represent the intersection between the inner wall of the artery and the planes (with normal *x*) containing the centroids of the two coils. (*a*) Scheme of the unit cell and (*b*) scheme of the quasi-periodic conditions. (Online version in colour.)

Bloch–Floquet quasi-periodicity conditions are imposed, as shown in figure 2*b*, and they are given by

2.6*a* $$\begin{array}{r} {\text{U}}_{a}(x+L_{a},y,z)={\text{U}}_{a}(x,y,z)\;e^{ikL_{a}}, \end{array}$$

2.6*b* $$\begin{array}{r} P_{f}(x+L_{a},y,z)=P_{f}(x,y,z),\;e^{ikL_{a}} \end{array}$$

2.6*c* $$\begin{array}{rl} \;\text{and}\;{\text{U}}_{s}(x+L_{a},y,z) & ={\text{U}}_{s}(x,y,z)\;e^{ikL_{a}}, \end{array}$$

where *L*_a_ is the length of the unit cell, *k* is the wavenumber, which is inversely proportional to the wavelength *λ*=2*π*/*k*, and (*x*,*y*,*z*) is a point in the elementary cell, including the boundary.

### (b) Definition of the three-dimensional geometries

The unit cell for the stented artery is composed of a hollow cylinder (representing the wall of the vessel), two zigzag-shaped coils (representing the stent pattern) and a cylindrical fluid domain enclosed by the hollow cylinder (representing the blood), as sketched in figure 2*a*. The artery wall is modelled as a three-dimensional solid, having a length *L*_a_ of 10 mm, a lumen diameter 2*R*_a_ of 7.3 mm and a thickness *h*_a_ of 0.7 mm. Therefore, the outer diameter 2*R*_*b*_ is equal to 8.7 mm and the average radius is equal to 4 mm, reproducing representative values available in the literature (see, for example, [1], Tab. 4.2, p. 187). The zigzag-shaped coils are characterized by eight crowns (16 segments) and are modelled as beams with a constant circular cross section (0.1 mm diameter). The distance between the opposite crowns is equal to one-third of the unit cell length (≈3.333 mm), whereas the distance between the centroids of the two coils is equal to half the unit cell length (5 mm), as indicated in figure 2*a* (on the left and right sides, respectively).

**Figure 3.**
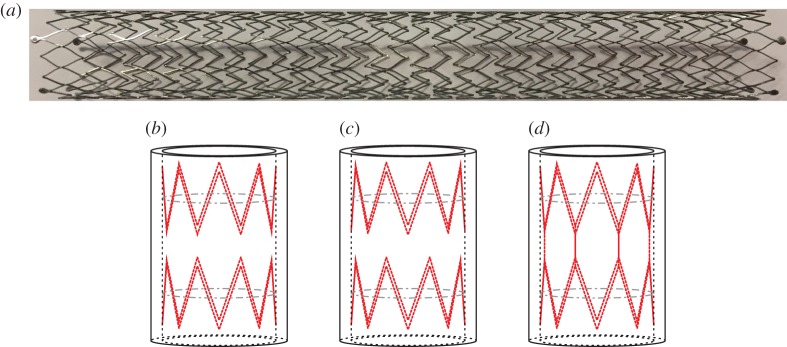
Examples of unit cell with different stent configurations employed in the numerical simulations. (*a*) Illustrates one among many stent geometries commercially available. (*b*–*d*) Represent different typical patterns for coils. (*a*) Example of a Cook silver stent structure, (*b*) unit cell with type A stents, (*c*) unit cell with type B stents and (*d*) unit cell with type A connected stents. (Online version in colour.)

The dynamic response of the stented artery with three different configurations of the coils, shown in figure 3, is investigated. In particular, the following cases are analysed: a symmetric unit cell (type A), where the coils are symmetric with respect to the middle cross section of the cell; a unit cell with unidirectional stents (type B), obtained by translation of the coils; a unit cell with connected type A stents, where some of the crowns are linked with additional beam elements (figure 3*d*).

### (c) Material parameters

Arteries are characterized by (nearly-)incompressible nonlinear behaviour, for which nonlinear elastic constitutive models (including the description as a heterogeneous material) are reported in the literature and their calibration based on experiments [36–41]. Nonlinear constitutive models for shape memory alloys are generally used to describe the behaviour of balloon-expandable and self-expanding stents [42,43], but some authors prefer to employ linear elastic constitutive models [44–46]. Nonlinear constitutive models for atherosclerotic tissue and restenosis are reported in [40,44,47].

The aim of this study is to identify possible pass-bands and stop-bands for the coupled system composed of the stented artery and the blood by means of the Bloch–Floquet approach. The components of the coupled system are modelled as linear elastic isotropic homogeneous materials. The elastic parameters for the artery tissue and the stents employed in this work are summarized in table 1, and they correspond to typical average values for the carotid artery [1,48] and for metals commonly used for stents [49]. The blood is modelled as an acoustic medium of bulk modulus *K*_f_=2.4 GPa and density *ρ*_f_=1050 kg m^−3^.

**Table 1.** Definition of the elastic properties of the materials employed in the simulations.

	materials	
properties	artery	stent
Young modulus	*E*_a_=800 kPa	*E*_s_=210 GPa
Poisson ratio	*ν*_a_=0.49	*ν*_s_=0.3
density	*ρ*_a_=1200 kg m^−3^	*ρ*_s_=7800 kg m^−3^

### (d) Dispersion curves

In this section, the dispersion properties of the Bloch–Floquet elastic waves propagating along the walls of the artery are discussed. The results are presented as dispersion curves in the wavenumber-frequency plane. The dispersion curves are even and 2*π*/*L*_a_ periodic functions. The interval [−*π*/*L*_a_,*π*/*L*_a_] is known as the *irreducible Brillouin zone* [50,51]. Owing to their symmetry and periodicity, the dispersion curves are illustrated for the range 0≤*k*≤*π*/*L*_a_.

**Figure 4.**
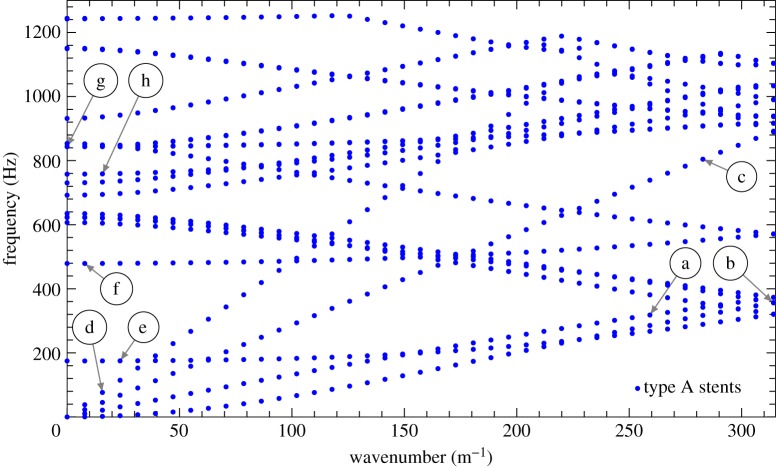
Dispersion curves in the wavenumber-frequency plane representing different vibration modes for the symmetric unit cell shown in figure 3*b*. The shapes of the deformation modes associated with the points highlighted with arrows are depicted in figure 1, where the same letters are used. (Online version in colour.)

**Figure 5.**
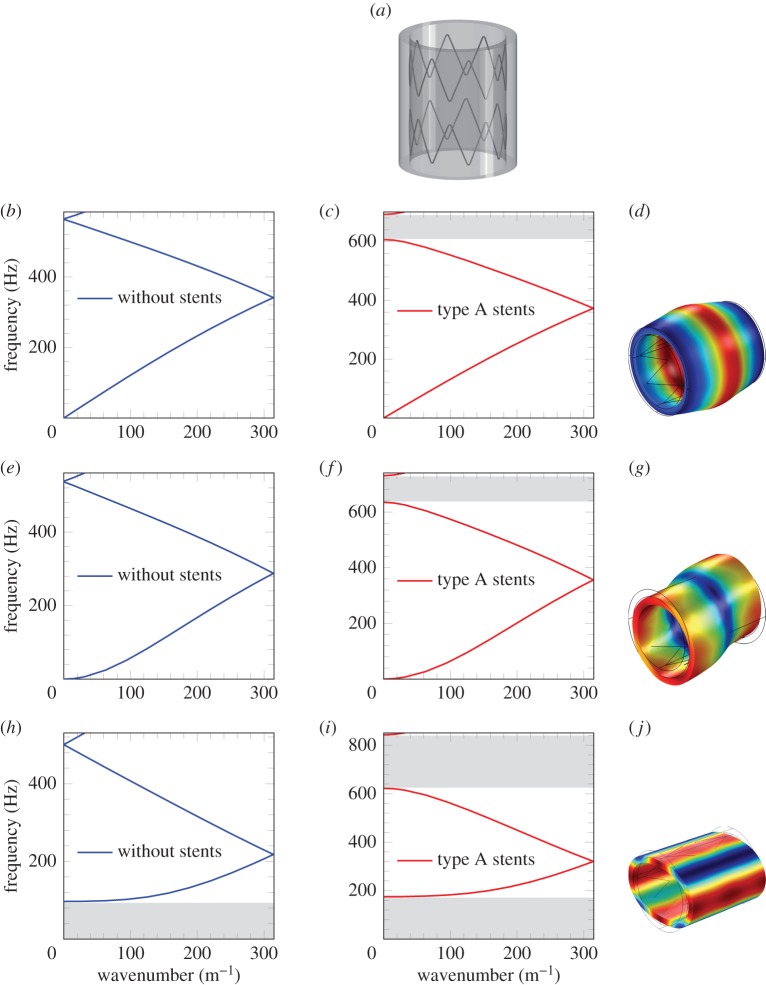
Dispersion curves for the axisymmetric mode (*b*–*d*), the flexural mode (*e*–*g*) and for the mode involving simple flattening of the wall (*h*–*j*), for the unit cell without stents and with type A stents. The shaded zones denote the stop-bands. (*a*) Unit cell with type A stents, (*b*) unit cell without stents, axisymmetric mode, (*c*) unit cell with type A stents, axisymmetric mode, (*d*) representation of the axisymmetric mode, (*e*) unit cell without stents, flexural mode, (*f*) unit cell with type A stents, flexural mode, (*g*) representation of the flexural mode, (*h*) unit cell without stents, mode involving simple flattening of the wall, (*i*) unit cell with type A stents, mode involving simple flattening of the wall and (*j*) representation of the mode involving simple flattening of the wall. (Online version in colour.)

**Figure 6.**
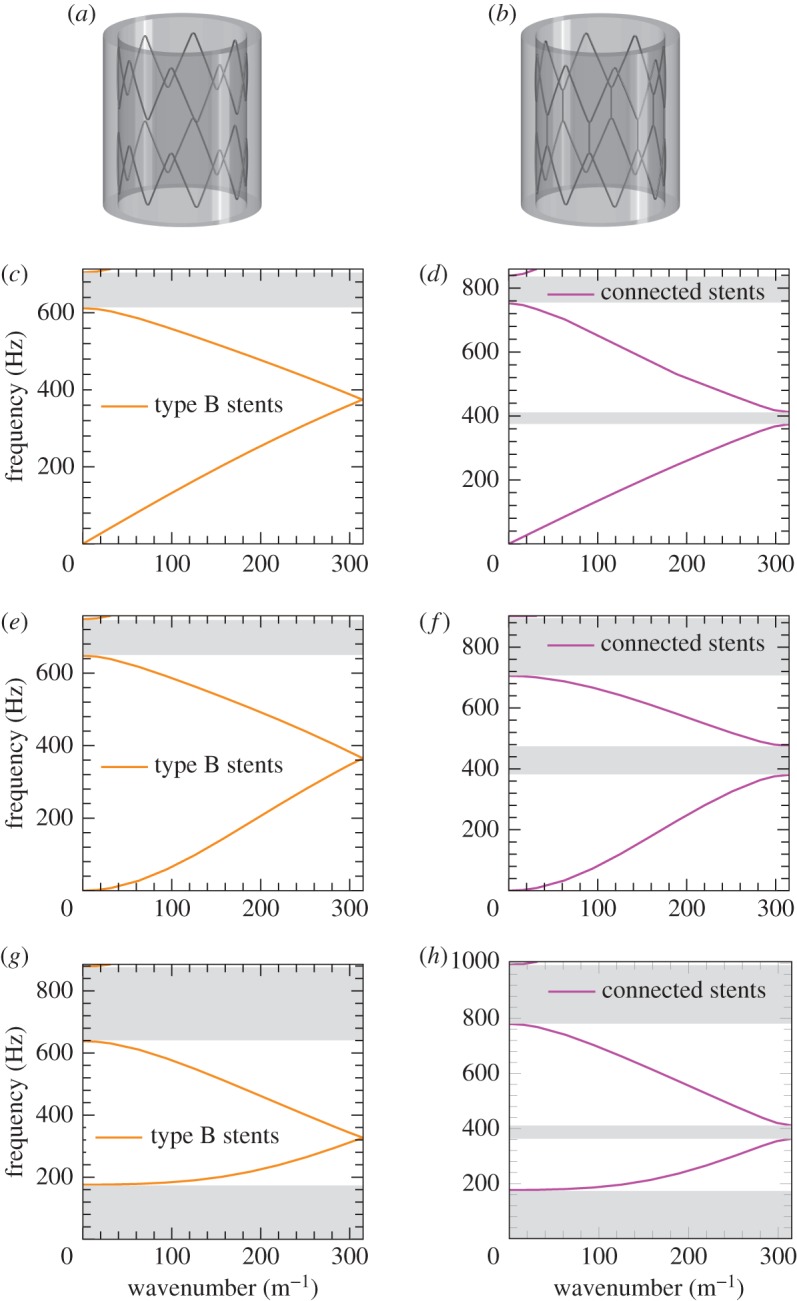
Comparison of the dispersion curves for the axisymmetric mode (*c*–*d*), the flexural mode (*e*–*f*) and for the mode involving simple flattening of the wall (*g*–*h*), for the unit cell with different types of stents. The shaded zones denote the stop-bands. (*a*) Unit cell with type B stents, (*b*) unit cell with connected type A stents, (*c*) unit cell with type B stents, axisymmetric mode, (*d*) unit cell with connected type A stents, axisymmetric mode, (*e*) unit cell with type B stents, flexural mode, (*f*) unit cell with connected type A stents, flexural mode, (*g*) unit cell with type B stents, mode involving simple flattening of the wall and (*h*) unit cell with connected type A stents, mode involving simple flattening of the wall. (Online version in colour.)

The dispersion diagrams presented in figures 4–6 identify *ω* as a multi-valued function of the Bloch–Floquet parameter *k*. The dispersion diagrams show the presence of stop-bands and standing waves in stented blood vessels. The stop-bands represent the intervals of frequencies for which only evanescent waveforms occur. Standing waves are characterized by zero group velocity and they are observed at the boundaries of stop-bands.

As a first step, the comparison between the case of a healthy artery and the case of an artery with type A stents (figure 3*b*) is provided. Subsequently, a comparison of the three types of stents depicted in figure 3 is presented to show the influence of the stent geometry on the dispersion properties of the system.

#### (i) Vibration modes: type A stents

Figure 4 presents the complete dispersion diagram for type A stents. The labels (*a*–*h*), which mark the individual dispersion curves in this figure, correspond to representative vibration modes, shown in figure 1. In particular, the four curves corresponding to modes (a–d) originate at 0 and are referred to as ‘acoustic’ dispersion curves. They are dominated by flexural motion (b), axially symmetric expansion/contraction deformation (a), torsional motion (c) and longitudinal motion (d). As the frequency is increased, the individual dispersion curves represent mixed-modes, which incorporate elastic deformations of different types, like the mode represented by curve (h). It should be noted that there are no common stop-bands. However, it is possible to investigate stop-band formation for each individual vibration mode separately, thus splitting figure 4 into a set of different curves representing different modes. In the following, a limited but representative number of vibration modes are discussed.

From figure 5*b*,*c*, it can be noted that the group velocities for the axisymmetric mode are slightly different, with an increase observed in the case of the stented artery. Furthermore, there are no stop-bands at low frequencies, and hence no blockages in transmission are observed. Conversely, at high frequencies, the appearance of a stop-band in the stented artery is noted. The same observations hold true for the flexural mode shown in figure 5*e*,*f*.

A different behaviour is detected for the mode involving simple flattening of the wall, shown in figure 5*h*,*i*. In fact, for the stented artery, the stop-band at low frequencies is much wider in comparison with the unstented artery, and an additional stop-band appears at high frequencies. The stop-band at low frequencies in figure 5*h* indicates that this mode cannot propagate within the typical frequency range of the blood vessels in human beings. When a type A stent is installed in the artery, this stop-band is wider, which prevents the propagation of the simple wall flattening mode within a larger frequency range, compared with the one that may have been activated without stents.

#### (ii) Vibration modes: comparison between different types of stents

From figures 5 and 6, it can be noted that the behaviour of the system with type A stents and type B stents is very similar. Connected type A stents show a slightly different group velocity at low frequencies compared with type A and B stents, and an additional stop-band appears between the first two dispersion curves associated with each mode. Furthermore, when connected type A stents are installed, the stop-band between the second and the third dispersion curves appears at higher frequencies compared with type A and B stents.

## 3. The cluster of stents

The cases analysed in the previous section are representative of common stent designs, where a single stent is installed in the artery. Periodicity and geometry of the reinforcements in the vessel can affect the dynamic response of the system in terms of wave propagation. Hence, the question of finding particular geometries and patterns inducing stop-bands at low-frequency regimes arises. Clinical experience shows that it is extremely rare for just one area of a diseased artery to be affected. It is not unusual for several small areas within one artery to have profound luminal reduction or a much longer segment affected. This raises the question as to whether it is better to put in multiple small stents or one long stent to treat several areas of disease at once. The case of multiple stents provides a different and very interesting pattern of the reinforcement of the vessel, linked to the periodicity of the structure, as shown by Papathanasiou *et al.* [29].

**Figure 7.**
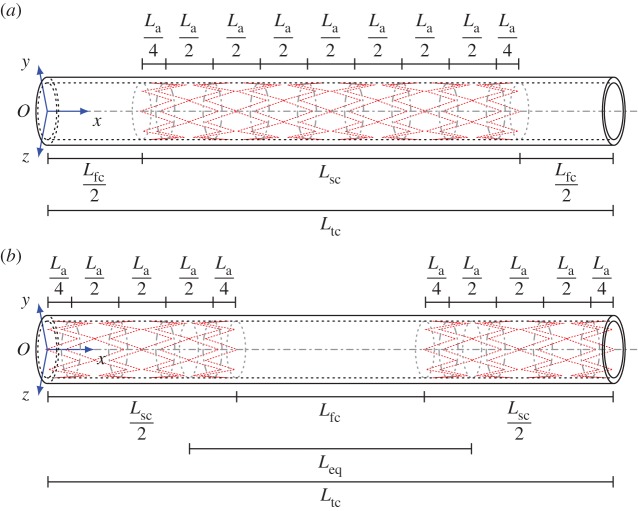
Geometry of the unit cell for the cluster of type A stents. The two versions are equivalent. (*b*) Shows clearly the spacing between the groups of coils, whereas (*a*) shows the length of the repeated stented zone. (*b*) Illustrates the determination of the length *L*_*eq*_ of the shell employed in the semi-analytical model shown in figure 9*a*. (*a*) Unit cell employed in the computations and (*b*) alternative unit cell, equivalent to (*a*). (Online version in colour.)

In this section, an in-depth investigation of the case in which more stents are installed in different sections of the arteries is provided for the three-dimensional model, thus generalizing the one-dimensional analysis by Papathanasiou *et al.* [29]. To this purpose, a unit cell is composed of a finite-length stent (denoted by *L*_sc_) and unstented section of the artery, which is then repeated periodically with a specific spacing *L*_fc_. The geometry of this system is shown in detail in figure 7, where type A stents are used. The total length *L*_tc_ of such a unit cell is equal to 60 mm and the length *L*_fc_ of the stent free zone is equal to 20 mm. Two equivalent unit cells can be employed, and these are shown in figure 7*a*,*b*.

### (a) Numerical model

**Figure 8.**
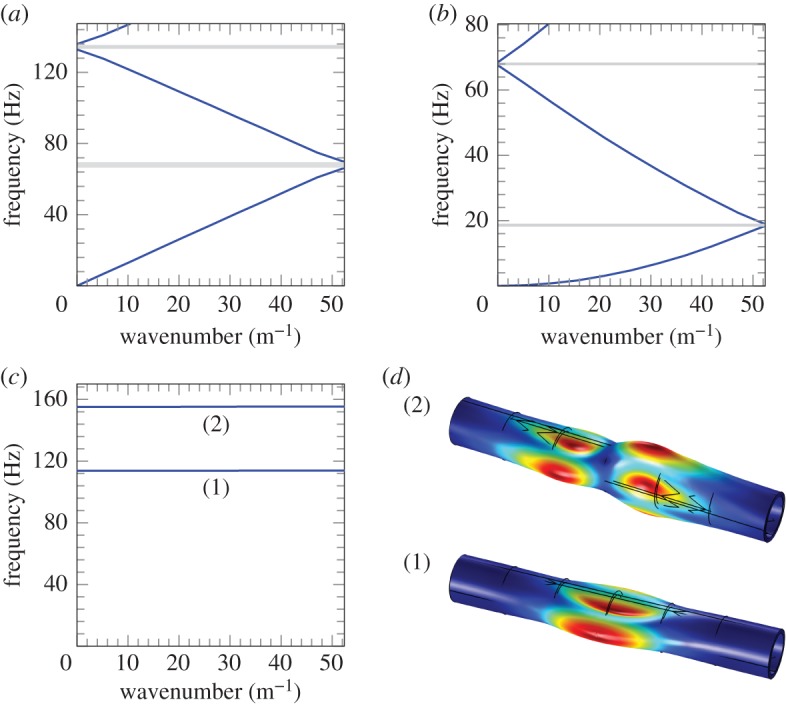
Dispersion curves for the cluster of stents. (*a*) Axisymmetric mode, (*b*) flexural mode, (*c*) modes corresponding to standing waves and (*d*) localized modes occurring within the arterial wall between the stents. (Online version in colour.)

The dispersion curves obtained from the Bloch–Floquet analysis are shown in figure 8. It can be noted that for the axisymmetric mode (figure 8*a*), there are two additional stop-bands appearing in the low-frequency regime, but the width of these stop-bands is much smaller compared with those determined for type A, type B and type A connected stents (figures 5*c* and 6*c*,*d*). Similarly, a narrow stop band appears in the low-frequency regime for the flexural mode (figure 8*b*), which is smaller than the first stop-band appearing for type A, type B and type A connected stents (figures 5*f* and 6*e*,*f*).

For the simple flattening of the wall (figure 8*c*), the dispersion curves have a slope close to zero, so they represent standing waves and/or waves with a very small group velocity. This means that energy is not transmitted through the system. The deformation modes corresponding to the standing waves, illustrated in figure 8*c*, show that the deformation occurs only within the zones separating the groups of stents (figure 8*d*). Therefore, the system behaves similarly to a simplified system composed of a fluid-filled cylinder with a length *L*_eq_=30 mm, indicated in figure 7*b*. Appropriate boundary conditions need to be applied at the end sections of this equivalent system. In particular, the deformation modes associated with the (quasi-)zero slope dispersion curves for the cluster of stents suggest the application of simply supported boundary conditions. The resonant frequencies of the equivalent system, corresponding to the standing waves for the cluster of stents, can be determined analytically as discussed in the next section.

Figure 8*d* shows that exponential localization and flattening of the arterial wall can occur in the unstented section. This can lead to a slight change in the shape of the lumen and subsequently influences the blood flow at higher frequencies.

### (b) Semi-analytical model

**Figure 9.**
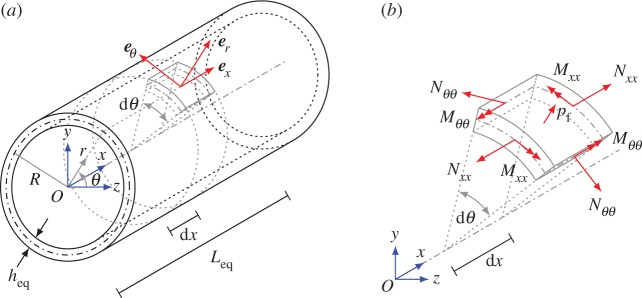
Scheme of the unit cell modelled as a cylindrical shell (*a*) and of the generalized stresses acting on its middle surface. (*b*) The determination of the length *L*_eq_ of the cylindrical shell is shown in figure 7*b*. (*a*) Cylindrical shell and its reference system and (*b*) generalized stresses acting on the middle surface of the shell. (Online version in colour.)

The frequencies corresponding to standing waves and to small group velocity waves, determined numerically in §3a, can be also estimated analytically by approximating the arterial wall as a finite elastic shell with the simply supported boundary conditions at the ends. The thin shell theory is employed here, together with the assumption of small displacements. The fluid exerts pressure on the artery wall. The equivalent cylindrical shell has thickness *h*_eq_, radius of the middle surface *R*_eq_ and length *L*_eq_. The reference system (*x*,*θ*,*r*) is depicted in figure 9, where the *x*-axis is the axis of the shell. The components of the displacement field ***u***=*u****e***_*x*_+*v****e***_*θ*_+*w****e***_*r*_ of the middle surface of the shell are aligned with the local *x*,*θ*,*r* directions, respectively.

#### (i) Framework of the thin shell theory

The equations of motion for a cylindrical thin shell have the form [52,53]

3.1*a* $$\begin{array}{r} \frac{\partial N_{xx}}{\partial x}+\frac{1}{R_{\mathrm{eq}}}\frac{\partial N_{x\theta }}{\partial \theta }=\rho_{a}h_{\mathrm{eq}}\frac{\partial^{2}u}{\partial t^{2}}, \end{array}$$

3.1*b* $$\begin{array}{r} \frac{\partial N_{x\theta }}{\partial x}+\frac{1}{R_{\mathrm{eq}}}\frac{\partial N_{\theta \theta }}{\partial \theta }+\frac{1}{R_{\mathrm{eq}}}\frac{\partial M_{x\theta }}{\partial x}+\frac{1}{R_{\mathrm{eq}}^{2}}\frac{\partial M_{\theta \theta }}{\partial \theta }=\rho_{a}h_{\mathrm{eq}}\frac{\partial^{2}v}{\partial t^{2}} \end{array}$$

3.1*c* $$\begin{array}{r} \;\text{and}\;\frac{\partial^{2}M_{xx}}{\partial x^{2}}+\frac{2}{R_{\mathrm{eq}}}\frac{\partial^{2}M_{x\theta }}{\partial x\partial \theta }+\frac{1}{R_{\mathrm{eq}}^{2}}\frac{\partial^{2}M_{\theta \theta }}{\partial \theta^{2}}-\frac{N_{\theta \theta }}{R_{\mathrm{eq}}}+f_{r}=\rho_{a}h_{\mathrm{eq}}\frac{\partial^{2}w}{\partial t^{2}}, \end{array}$$

where the external load *f*_*r*_ represents the fluid pressure at the fluid–solid interface, whereas the generalized stresses are given by

3.2*a* $$(N_{xx},N_{\theta \theta },N_{x\theta })=\int_{-h_{\mathrm{eq}}/2}^{h_{\mathrm{eq}}/2}(\sigma_{xx},\sigma_{\theta \theta },\sigma_{x\theta })\;dr$$

and

3.2*b* $$(M_{xx},M_{\theta \theta },M_{x\theta })=\int_{-h_{\mathrm{eq}}/2}^{h_{\mathrm{eq}}/2}(\sigma_{xx},\sigma_{\theta \theta },\sigma_{x\theta })r\;dr.$$

The constitutive equations for a linear elastic isotropic homogeneous material, relating the stress tensor ***σ*** in equation (3.2) to the strain tensor ***ε***, have the form

3.3 $$\left(\begin{array}{c} \sigma_{xx}\\ \sigma_{\theta \theta }\\ \sigma_{x\theta } \end{array}\right)=\left(\begin{array}{ccc} Q_{11} & Q_{12} & 0\\ Q_{12} & Q_{22} & 0\\ 0 & 0 & Q_{66} \end{array}\right)\;\left(\begin{array}{c} \varepsilon_{xx}\\ \varepsilon_{\theta \theta }\\ \varepsilon_{x\theta } \end{array}\right).$$

The non-zero components of the elastic matrix ***Q*** are

3.4 $$Q_{11}=Q_{22}=\frac{E_{a}}{1-\nu_{a}^{2}},\;Q_{12}=\frac{E_{a}\nu_{a}}{1-\nu_{a}^{2}}\;\mathrm{and}\;Q_{66}=\frac{Q_{11}-Q_{12}}{2}=\frac{E_{a}}{2(1+\nu_{a})},$$

where *E*_a_ and *ν*_a_ are the Young modulus and the Poisson ratio of the shell’s material (the artery), respectively. Following Love’s theory, the components of the strain tensor ***ε*** introduced in equation (3.3) are defined in terms of the displacement field ***u*** as

3.5*a* $$\begin{array}{r} \varepsilon_{xx}=\frac{\partial u}{\partial x}-\frac{\partial^{2}w}{\partial x^{2}}r, \end{array}$$

3.5*b* $$\begin{array}{r} \varepsilon_{\theta \theta }=\frac{1}{R_{\mathrm{eq}}}\left(\frac{\partial v}{\partial \theta }+w\right)+\frac{1}{R_{\mathrm{eq}}^{2}}\left(\frac{\partial v}{\partial \theta }-\frac{\partial^{2}w}{\partial \theta^{2}}\right)\;r \end{array}$$

3.5*c* $$\begin{array}{r} \;\text{and}\;\varepsilon_{x\theta }=\frac{\partial v}{\partial x}+\frac{1}{R_{\mathrm{eq}}}\frac{\partial u}{\partial \theta }+\frac{1}{R_{\mathrm{eq}}}\left(\frac{\partial v}{\partial x}-2\frac{\partial^{2}w}{\partial x\partial \theta }\right)\;r. \end{array}$$

Substituting equations (3.2)–(3.5) into equation (3.1) yields the following form for the equations of motion:

3.6 $$\left(\begin{array}{ccc} L_{11} & L_{12} & L_{13}\\ L_{21} & L_{22} & L_{23}\\ L_{31} & L_{32} & L_{33} \end{array}\right)\;\left(\begin{array}{c} u\\ v\\ w \end{array}\right)=\left(\begin{array}{c} 0\\ 0\\ -f_{r} \end{array}\right),$$

where *L*_*ij*_ (*i*,*j*=1,2,3) are the differential operators with respect to *x* and *θ*, given by

3.7*a* $$\begin{array}{r} L_{11}=\frac{E_{a}h_{\mathrm{eq}}}{\left(1-\nu_{a}^{2}\right)}\frac{\partial^{2}}{\partial x^{2}}+\frac{E_{a}h_{\mathrm{eq}}}{2(1+\nu_{a})R_{\mathrm{eq}}^{2}}\frac{\partial^{2}}{\partial \theta^{2}}-\rho_{a}h_{\mathrm{eq}}\frac{\partial^{2}}{\partial t^{2}}, \end{array}$$

3.7*b* $$\begin{array}{r} L_{12}=L_{21}=\frac{E_{a}h_{\mathrm{eq}}}{2(1-\nu_{a})R_{\mathrm{eq}}}\frac{\partial^{2}}{\partial x\partial \theta }, \end{array}$$

3.7*c* $$\begin{array}{r} L_{13}=-L_{31}=\frac{\nu_{a}E_{a}h_{\mathrm{eq}}}{(1-\nu_{a}^{2})R_{\mathrm{eq}}}\frac{\partial }{\partial x}, \end{array}$$

3.7*d* $$\begin{array}{r} L_{22}=\frac{E_{a}h_{\mathrm{eq}}}{2(1+\nu_{a})}\left(1+\frac{h_{\mathrm{eq}}^{2}}{12R_{\mathrm{eq}}^{2}}\right)\frac{\partial^{2}}{\partial x^{2}}+\frac{E_{a}h_{\mathrm{eq}}}{(1-\nu_{a}^{2})R_{\mathrm{eq}}^{2}}\left(1+\frac{h_{\mathrm{eq}}^{2}}{12R_{\mathrm{eq}}^{2}}\right)\frac{\partial^{2}}{\partial \theta^{2}}-\rho_{a}h_{\mathrm{eq}}\frac{\partial^{2}}{\partial t^{2}}, \end{array}$$

3.7*e* $$\begin{array}{r} L_{23}=-L_{32}=\frac{E_{a}h_{\mathrm{eq}}}{(1-\nu_{a}^{2})R_{\mathrm{eq}}^{2}}\frac{\partial }{\partial \theta }-\frac{E_{a}h_{\mathrm{eq}}^{3}}{12(1-\nu_{a}^{2})R_{\mathrm{eq}}^{2}}\frac{\partial^{3}}{\partial x^{2}\partial \theta }-\frac{E_{a}h_{\mathrm{eq}}^{3}}{12(1-\nu_{a}^{2})R_{\mathrm{eq}}^{4}}\frac{\partial^{3}}{\partial \theta^{3}} \end{array}$$

3.7*f* $$\begin{array}{r} \;\text{and}\;L_{33}=-\frac{E_{a}h_{\mathrm{eq}}^{3}}{12(1-\nu_{a}^{2})}{\left(\frac{\partial^{2}}{\partial x^{2}}+\frac{1}{R_{\mathrm{eq}}^{2}}\frac{\partial^{2}}{\partial \theta^{2}}\right)}^{2}-\frac{E_{a}h_{\mathrm{eq}}}{(1-\nu_{a}^{2})R_{\mathrm{eq}}^{2}}-\rho_{a}h_{\mathrm{eq}}\frac{\partial^{2}}{\partial t^{2}}. \end{array}$$

The fluid is modelled as an acoustic medium, hence the equations of motion of the fluid can be expressed in the cylindrical coordinate system (*x*,*θ*,*r*) as

3.8 $$\frac{1}{r}\frac{\partial }{\partial r}\left(r\frac{\partial p_{f}}{\partial r}\right)+\frac{1}{r^{2}}\frac{\partial^{2}p_{f}}{\partial \theta^{2}}+\frac{\partial^{2}p_{f}}{\partial x^{2}}=\frac{1}{C_{f}^{2}}\frac{\partial^{2}p_{f}}{\partial t^{2}},$$

where *p*_f_ is the fluid pressure and *C*_f_ is the speed of sound in the fluid (Cf=Kf/ρf;;).

#### (ii) Time-harmonic regime

In the framework of the time-harmonic regime, the displacement field ***u*** of the shell can be expressed in the form of a travelling wave, associated with an axial wavenumber *k* and circumferential mode number *n*. The expression of the displacement field ***u*** is

3.9*a* $$\begin{array}{r} u(x,\theta ,t)=U\;e^{ikx}\sin(n\theta )\;\cos(\omega t), \end{array}$$

3.9*b* $$\begin{array}{r} v(x,\theta ,t)=V\;e^{ikx}\;\cos(n\theta )\;\cos(\omega t) \end{array}$$

3.9*c* $$\begin{array}{r} \;\mathrm{and}\;w(x,\theta ,t)=W\;e^{ikx}\sin(n\theta )\;\cos(\omega t), \end{array}$$

where *ω* is the radian frequency, and *U*,*V*,*W* are the wave amplitudes in the *x*,*θ*,*r* directions, respectively. The associated form of the acoustic pressure field is expressed as

3.10 $$p_{f}=P_{f}\;e^{ikx}\;\cos(n\theta )J_{n}(k_{r}r)\;\cos(\omega t),$$

where *P*_f_ is the pressure amplitude of the acoustic fluid, *k*_*r*_ is the radial wavenumber and *J*_*n*_(*k*_*r*_*r*) is the Bessel function of the first kind of order *n*. The radial wavenumber is related to the axial wavenumber by the relation

3.11 $$k_{r}=\sqrt{\frac{\omega^{2}}{C_{f}^{2}}-k^{2}}.$$

#### (iii) Approximation of the trapped waveforms

The fluid–solid interaction is taken into account by imposing the following boundary condition in terms of equivalence between the acceleration of the fluid and the shell:

3.12 $${\left.\frac{\partial^{2}w}{\partial t^{2}}\right|}_{r=R_{\mathrm{eq}}}={\left.\frac{\partial v_{f}}{\partial t}\right|}_{r=R_{\mathrm{eq}}}=-\frac{1}{\rho_{f}}\frac{\partial p_{f}}{\partial r}.$$

Substituting equations (3.9c) and (3.10) into the boundary conditions (3.12) yields the pressure amplitude *P*_f_ of the acoustic fluid in the form

3.13 $$P_{f}=\frac{\omega^{2}\rho_{f}}{k_{r}J_{n}^{\prime }(k_{r}r)}W.$$

The displacement field (3.9) and the pressure amplitude (3.13) can be substituted into the equations of motion (3.6), so that the equations of motion of the coupled system can be written as

3.14 $$\left(\begin{array}{ccc} C_{11} & C_{12} & C_{13}\\ C_{21} & C_{22} & C_{23}\\ C_{31} & C_{32} & C_{33} \end{array}\right)\;\left(\begin{array}{c} U\\ V\\ W \end{array}\right)=\left(\begin{array}{c} 0\\ 0\\ 0 \end{array}\right),$$

where the elements *C*_*ij*_ are given by

3.15*a* $$\begin{array}{r} C_{11}=\frac{E_{a}h_{\mathrm{eq}}}{1-\nu_{a}^{2}}k^{2}+\frac{E_{a}h_{\mathrm{eq}}}{2(1+\nu_{a})R_{\mathrm{eq}}^{2}}n^{2}-\rho_{a}h_{\mathrm{eq}}\omega^{2}, \end{array}$$

3.15*b* $$\begin{array}{r} C_{12}=i\frac{E_{a}h_{\mathrm{eq}}}{2(1-\nu_{a})R_{\mathrm{eq}}}nk=-C_{21}, \end{array}$$

3.15*c* $$\begin{array}{r} C_{13}=-i\frac{E_{a}h_{\mathrm{eq}}\nu_{a}}{(1-\nu_{a}^{2})R_{\mathrm{eq}}}k=-C_{31}, \end{array}$$

3.15*d* $$\begin{array}{r} C_{22}=\left(1+\frac{h_{\mathrm{eq}}^{2}}{12R_{\mathrm{eq}}^{2}}\right)\;\left[\frac{E_{a}h_{\mathrm{eq}}}{(1-\nu_{a}^{2})R_{\mathrm{eq}}^{2}}n^{2}+\frac{E_{a}h_{\mathrm{eq}}}{2(1+\nu_{a})}k^{2}\right]-\rho_{a}h_{\mathrm{eq}}\omega^{2}, \end{array}$$

3.15*e* $$\begin{array}{r} C_{23}=C_{32}=-\frac{E_{a}h_{\mathrm{eq}}n}{(1-\nu_{a}^{2})R_{\mathrm{eq}}^{2}}\left[\frac{h_{\mathrm{eq}}^{2}}{12}\left(k^{2}+\frac{n^{2}}{R_{\mathrm{eq}}^{2}}\right)+1\right] \end{array}$$

3.15*f* $$\begin{array}{r} \;\text{and}\;C_{33}=\frac{E_{a}h_{\mathrm{eq}}}{\left(1-\nu_{a}^{2}\right)}\left[\frac{1}{R_{\mathrm{eq}}^{2}}+\frac{h^{2}}{12}{\left(\frac{n^{2}}{R_{\mathrm{eq}}^{2}}-k^{2}\right)}^{2}\right]-\rho_{a}h_{\mathrm{eq}}\omega^{2}+f_{r}, \end{array}$$

and the fluid loading term takes the form

3.16 $$f_{r}=-\frac{\rho_{f}}{k_{r}}\frac{J_{n}(k_{r}R_{\mathrm{eq}})}{J_{n}^{\prime }(k_{r}R_{\mathrm{eq}})}\omega^{2}.$$

The assumption of simply supported ends (at *x*=0 and *x*=*L*_eq_) yields the following boundary conditions

3.17 $$v=0,\;w=0,\;N_{xx}=0,\;M_{xx}=0,\;\text{at}\;x=0\;\text{and}\;x=L_{\mathrm{eq}}.$$

In order to satisfy the boundary conditions (3.17), the axial wavenumber *k* is taken as

3.18 $$k=\frac{\pi m}{L_{\mathrm{eq}}}.$$

For (3.14) to have non-trivial solutions, the determinant of ***C*** must be equal to 0, so that the characteristic equation takes the form

3.19 F(m,n,ω)=0.

#### (iv) Frequency comparison for the simplified structure

The term ‘simplified structure’ is used here for a finite section of the blood vessel, of length *L*_eq_ between the stents. To observe the trapped waveforms, finite-element analysis is also performed for the case when the appropriate boundary conditions are set at the edges of the finite section. These computations are compared with the results obtained from the semi-analytical shell model described above.

Equation (3.19) is used to obtain the natural frequencies of the fluid-filled shell that approximate the frequencies corresponding to the dispersion curves for the cluster of stents with zero or small slope. The second column of table 2 summarizes the first three frequencies for standing waves evaluated by means of the semi-analytical model. The results are compared with the frequencies obtained through two finite-element models of the simplified structure described in this section. In particular, in the first model the artery is modelled as a three-dimensional solid, whereas in the second model the artery is modelled as a shell.

**Table 2.** Comparative results in terms of frequency between the semi-analytical model and the finite-element analysis for the determination of the standing waves within the cluster of stents. In the semi-analytical model, the results refer to the case *m*=2 assuming length *L*_eq_=30 mm, radius *R*_eq_=4 mm and thickness *h*_eq_=0.7 mm. In the first column, *n* denotes the circumferential mode number.

		finite structure		
	Bloch–Floquet	semi-analytical	simplified structure	simplified structure
*n*	approach (Hz)	model (Hz)	(solid) (Hz)	(shell) (Hz)
1	113.82	120.94	107.23	121.01
2	155.08	156.29	146.61	160.35
3	206.77	225.96	216.98	226.29

The first column in table 2 corresponds to standing waves, with quasi-periodicity boundary conditions set on the edges of the elementary cell. The third column corresponds to a finite hollow cylinder, whose displacements at the edge boundaries are equal to zero. The fourth column is produced from the finite-element computations for the elastic shell in the framework of the Kirchhoff–Love shell theory, with the simply supported edges of the finite section of the blood vessel.

A comparison between the semi-analytical model and the simplified structure where the artery is modelled as a shell (columns 2 and 4 of table 2) shows that there is a good agreement between the two models.

On the other hand, the model in which the artery is treated as a three-dimensional solid shows a small difference (compared with the approximation based on the shell theory) in the values of the frequencies corresponding to standing waves. This difference is associated with the choice of the fixed displacement boundary conditions at the edges of the thin-walled solid used in the calculations. Furthermore, it can be noted that the values obtained from the finite three-dimensional structure are closer to those obtained from the Bloch–Floquet analysis for the cluster of stents (column 1 of the table 2). The values of the frequencies estimated using the semi-analytical model provide a good approximation for the standing waves frequency characterized by exponential localization within the unstented arterial wall.

## 4. Transmission problem

**Figure 10.**
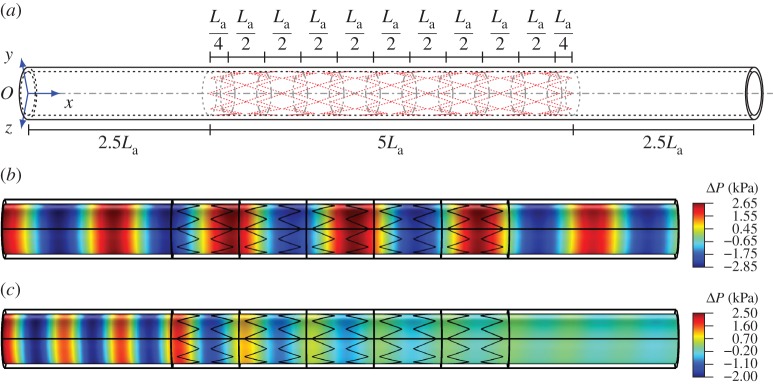
Scheme of the finite-length structure employed in frequency response analysis for type A stents. The system is based on the repetition of five unit cells of type A stents illustrated in figure 3*b*, where two sections of unstented artery are present at the left end and at the right end. The pressure field is shown in (*b*) and (*c*). (*a*) Scheme of the finite-length structure, (*b*) map of the pressure within a pass-band at 400 Hz and (*c*) map of the pressure within a stop-band at 650 Hz. (Online version in colour.)

**Figure 11.**
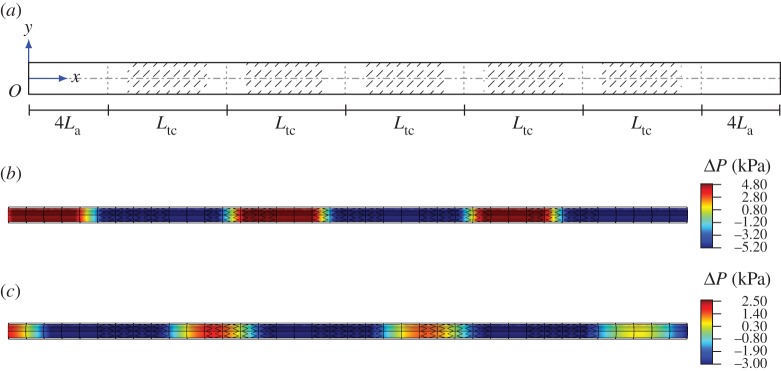
Scheme of the finite-length structure employed in frequency response analysis. The system is based on the repetition of five unit cells of clusters of type A stents illustrated in figure 7*a*, where two portions of artery without stents are positioned at the left end and at the right end. The pressure field is shown in (*b*) and (*c*). (*a*) Scheme of the finite-length structure, (*b*) map of the pressure within a pass-band at 61 Hz and (*c*) map of the pressure within a stop-band at 68 Hz. (Online version in colour.)

In this section, the transmission problem for the single stent and for the cluster of stents in a finite-length artery is presented. The material properties employed for the models discussed in this section are reported in table 1. In both cases, a pressure with amplitude *p*_0_=2.6 kPa≈20 *mmHg* is applied at *x*=0, and it generates a wave which propagates from the left-hand side to the right-hand side of the finite-length system (figures 10*a* and 11*a*). Zero displacement boundary conditions are applied to the end sections of the artery.

The analysis is performed in the following way. In the first step, the response of the system is investigated within the frequency range corresponding to a pass-band. In the second step, the investigation is restricted to a frequency range corresponding to the first stop-band of the system related to the axisymmetric mode, which was determined in §2d (figure 5*c*).

### (a) Single type A stent

The geometry for the case of a single type A stent is represented in figure 10*a*, where the total length of the artery is equal to 100 mm. A type A stent composed of 10 coils spaced by a distance *L*_a_/2=5 mm is inserted in the middle of the artery, so that the left end and the right end are not supported by stents.

From figure 10*b*, it can be noted that within the pass-band regime for the axisymmetric mode, waves can propagate without dissipation of energy and no reflection is detected; in this figure, the pressure field at the frequency of 400 Hz is shown. Conversely, waves having a frequency within the stop-band regime for the axisymmetric mode (ranging from 606.9 to 692.1 Hz) cannot propagate through the system (figure 10*c*).

### (b) Cluster of type A stents

The geometry for the case of a cluster of type A stents is represented in figure 11*a*, where the total length of the artery is equal to 380 mm. The system is composed of five unit cells (each of length *L*_tc_=60 mm) described in figure 7*a*, where type A stents are employed. Additional sections of stent-free artery (length equal to 4*L*_a_) are present at the left and at the right ends of the assembly of unit cells.

Similar to the case of a single stent, figure 11*b* shows that waves can propagate without dissipation within the pass-band regime for the axisymmetric mode and no reflection is detected; in this figure, the effect of a wave of frequency 61 Hz is illustrated. Conversely, waves having a frequency inside the stop-band regime cannot propagate through the system (figure 11*c*). In the latter case, where the frequency of the wave is equal to 68 Hz, the decrease in pressure amplitude is less evident because the stop-band width for the axisymmetric mode of this system (ranging from 66.3 to 69.7 Hz) is narrower compared with that of the single stent (606.9 to 692.1 Hz).

## 5. Response of the system in the transient regime

The pulsatile nature of flow is different in the arterial tree depending upon the anatomical position and the resistance in its draining arterial bed (organs supplied). The vessel calibre differs depending upon anatomical location and the blood flow required at times of activity or rest. The arteries are also subjected to the effects of human activities including low-frequency walking or running, as well as higher frequencies such as riding in vehicles. This means that evolution of the pulsatile flow can play an important role in the behaviour of a stented artery. Therefore, a further investigation in the framework of the transient regime is required to complete the dynamic analysis of the stented artery. This can improve the understanding of failure when changes on the wave propagation and stress occur, and can help to explain the observed tissue reactions to their placements [54,55].

### (a) Computational transient framework

A computational model is developed for the analysis of a finite-length artery in the transient regime. In the first step, transient regime analysis is performed for an idealized straight artery without stents; in the second step, the analysis is performed for the same artery where type B stents are installed, and a comparison of the results is provided.

The artery is modelled as a hollow cylinder, whose length is 100 mm, with inner and outer diameters equal to 7.3 and 8.7 mm, respectively. Zero displacement boundary conditions are applied to the end sections of the artery. In the case of a stented artery, the vessel is reinforced with 10 coils, whose spacing is equal to 5 mm, placed in the centre of the system, thus providing the geometry represented in figure 10*a*, where type A stents are replaced with type B stents. Differently from the previous section of this text, the stents are modelled as three-dimensional solids with square cross section (0.1 mm×0.1 mm). Therefore, equations of motion (2.1) are used for the artery and for the stents, whereas the complete Navier–Stokes equations are employed to model the fluid (blood). The Navier–Stokes equations are written as

5.1 $$\frac{\partial {\text{v}}_{f}}{\partial t}+({\text{v}}_{f}\cdot \nabla ){\text{v}}_{f}+\frac{\nabla p_{f}}{\rho_{f}}-\frac{\mu_{f}}{\rho_{f}}\nabla^{2}{\text{v}}_{f}=\text{0},$$

where ***v***_f_ is the velocity field of the fluid and *μ*_f_ is the dynamic viscosity.

#### (i) Material properties and boundary conditions

Consistent with the analyses reported in the previous sections, linear elastic isotropic homogeneous materials are employed for the artery and the stents. The material properties of the artery and of the stents are reported in table 1. The blood is modelled as a viscous incompressible fluid of density 1050 kg m^−3^ and of dynamic viscosity 0.003 Pa s.

The continuity of the displacements and tractions between the stents and the artery is applied at the interface corresponding to the external surface of the stent and the inner surface of the artery. It is assumed that the inlet and the outlet surfaces of the fluid are positioned at *x*=0 mm and at *x*=100 mm, respectively.

Full coupling between the fluid and the structure is taken into account. Coupling is provided by means of condition (2.3), which involves a relation between the fluid pressure and the stress in the artery, together with the following relation between the velocity of the fluid and the displacement of the artery

5.2 $${\text{v}}_{f}=\frac{\partial {\text{u}}_{a}}{\partial t},$$

representing the no-slip boundary condition for the viscous fluid.

#### (ii) Initial conditions

At the initial time *t*=0 *s*, the whole system is at rest. Furthermore, a velocity field ***v***_0_ is applied to the fluid at the inlet. The magnitude of the initial inlet mean velocity *v*_*m*,0_ is equal to 0.2 *m* *s*^−1^ and laminar flow regime is assumed, whereas the outlet pressure is assumed to equal zero. In a first phase, representing the initialization of the flow, the inlet velocity is assumed constant (namely, ***v***(*t*)=***v***_0_, hence *v*_*m*_(*t*)=*v*_*m*,0_) in order to reach the steady-state condition at a certain time *t*_0_. During this phase, the velocity profile of the flow takes the form of a circular paraboloid, corresponding to the Poiseuille flow. The maximum velocity of this profile is equal to 0.4 *m* *s*^−1^. A distribution *p*_*c*_, representing the shape of a circular paraboloid, is applied to the inlet velocity field in order to facilitate the system to reach the steady-state regime. In particular, for the system depicted in figure 10*a,* the distribution *p*_*c*_ is expressed as

5.3 $$p_{c}=1-\frac{y^{2}+z^{2}}{R_{a}^{2}},$$

where *R*_a_ is the inner radius of the artery, *y* and *z* are the Cartesian coordinates describing the inlet surface at *x*=0 mm. Therefore, the initial inlet velocity field can be expressed as

5.4 $${\text{v}}_{0}=2v_{m,0}p_{c}{\text{e}}_{x},$$

where ***e***_*x*_ is the unit vector oriented along the *x*-axis, which is the axis of the artery.

#### (iii) Pulsating flow

After reaching the steady-state regime, pulsating flow inlet conditions are assumed (***v***(*t*)≠***v***_0_ for *t*≥*t*_0_) as follows. An idealized pulsation representing the variation of inlet velocity is expressed in terms of Gaussian approximations and corresponds to 4 beats per second. This beat-rate, corresponding to 240 bpm, is representative of patients affected by tachycardia. It should be noted that the interest is focused on some of the frequencies composing the pulsation signal, which might be attenuated by the stent structure. The idealized pulsation is chosen as a continuous function approximating the velocity profile in an artery (see for instance [56], Fig. 1).

In the time-amplitude plane, the shape of this pulsation profile is flat (corresponding to the initial inlet mean velocity *v*_*m*,0_) for approximately two-thirds of the period, whereas, in the remaining part of the period, there is the variation from *v*_*m*,0_ to the maximum peak (assumed to be equal to 1.1*v*_*m*,0_), and back again to *v*_*m*,0_ (the same holds for the velocity field), thus representing the beat.

The pulsation is defined by a smooth function, which is periodically extended for all values of the time variable *t*. On a fixed interval, this function is approximated by a linear combination of ‘shifted’ Gaussians. In particular, it can be noted that for a sufficiently large *N* the function

5.5 $$\text{Const}\;\sum_{k=-2N}^{2N}\;e^{-{\left(t-k\right)}^{2}},$$

approximates a function, which is constant in the interval *t*∈(−*N*/2,*N*/2), and decays exponentially fast when |*t*|>2*N*. This function is infinitely differentiable and is fully suitable for approximating smooth pulses in the transient model. The desired pulsation is obtained by means of a distribution *b*(*t*) applied to the constant velocity field ***v***_0_. The expression employed for the pulsating inlet velocity profile is

**Figure 12.**
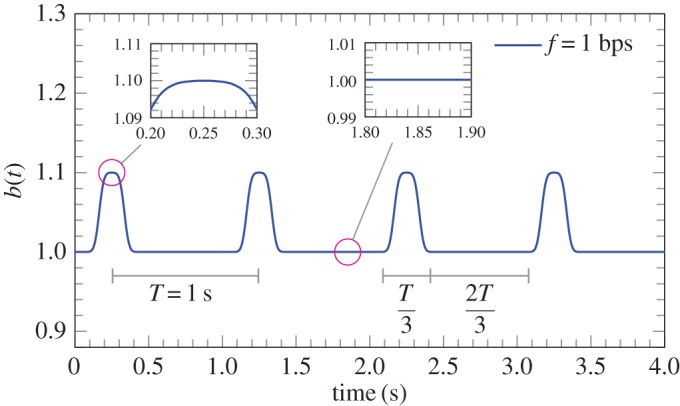
Graph of the function *b*(*t*) expressed by (5.6) assuming *A*_0_=1, Δ*A*=0.1 and *n*=20. (Online version in colour.)

5.6 $$b(t)=A_{0}+\Delta A{\left(\sum_{k=-n}^{n}\exp\left(-\frac{k^{2}}{72}\right)\right)}^{-1}\sum_{j=0}^{m}\sum_{k=-n}^{n}\exp\left\{-\frac{1}{72}{\left[240\left(ft-j-\frac{1}{4}\right)-k\right]}^{2}\right\},$$

where *A*_0_ is the initial amplitude (unit value is assumed), Δ*A* is the variation of the amplitude (assumed equal to 0.1), *f* is the number of beats per second, *m* denotes the total number of beats and *n* corresponds to the number of the series elements approximating the flat zone (*n*=20 provides a good approximation and has been used in the simulations). The function *b*(*t*) in equation (5.6) is plotted in figure 12. The inlet velocity field is expressed as

5.7 $$\text{v}(t)=\left\{\begin{array}{ll} {\text{v}}_{0} & t<t_{0},\\ b(t){\text{v}}_{0} & t\geq t_{0}. \end{array}\right.$$

Equation (5.7) provides the inlet condition that is applied in the computational model for the whole duration of the analysis.

The inlet velocity field ***v***(*t*) provided in equation (5.7) is smooth in the time domain (*b* is infinitely differentiable with respect to time) and therefore more suitable for transient analysis computations. The analysis is performed for a total of 30 beats after the initialization of the flow, which takes place at approximately 2.8 s.

The velocity field ***v***(*t*) can potentially promote different vibration modes of the system, because it includes several harmonics. From the spectral analysis of the distribution *b*(*t*), it can be noted that the amplitudes of the harmonics with a frequency higher than 32 Hz are already one-tenth of the amplitude of the first harmonic. Similarly, the amplitudes of higher harmonics having a frequency above 60 Hz are already two orders of magnitude below that of the first harmonic. Hence, the inlet velocity field excites a broad range of frequencies, although only a few of them can be considered in practice, because the effect of the others becomes negligible.

In the case of a pulsation corresponding to 3 Hz but with a variation of amplitude Δ*A* equal to 0.5, the spectral analysis shows that harmonics having a frequency above 40 Hz are nearly two orders of magnitude below that of the first harmonic.

### (b) Computations of the fluid velocity and elastic deformation of the blood vessel

**Figure 13.**
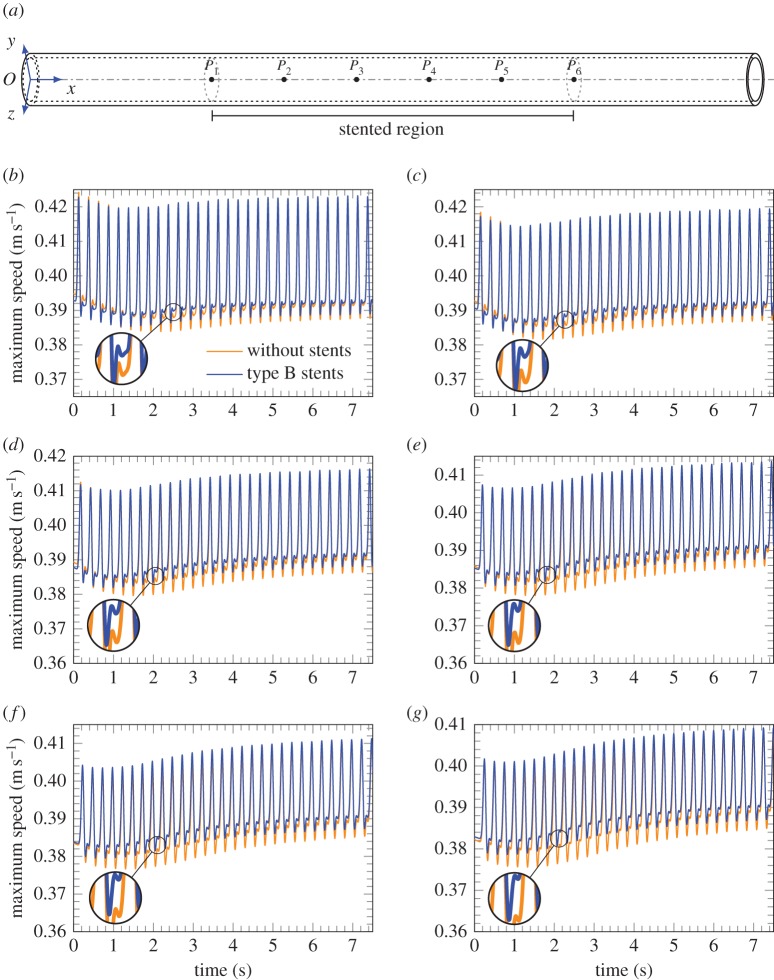
Evolution of the maximum fluid velocity at different points along the axis of the unstented artery and comparison with the case of a stented artery at 240 bpm, where the variation of amplitude Δ*A* is assumed equal to 0.1. (*a*) Scheme of the points in which the maximum fluid velocity is measured, (*b*) point *P*_1_ at *x*=25 mm, (*c*) point *P*_2_ at *x*=35 mm, (*d*) point *P*_3_ at *x*=45 mm, (*e*) point *P*_4_ at *x*=55 mm, (*f*) point *P*_5_ at *x*=65 mm and (*g*) point *P*_6_ at *x*=75 mm. (Online version in colour.)

**Figure 14.**
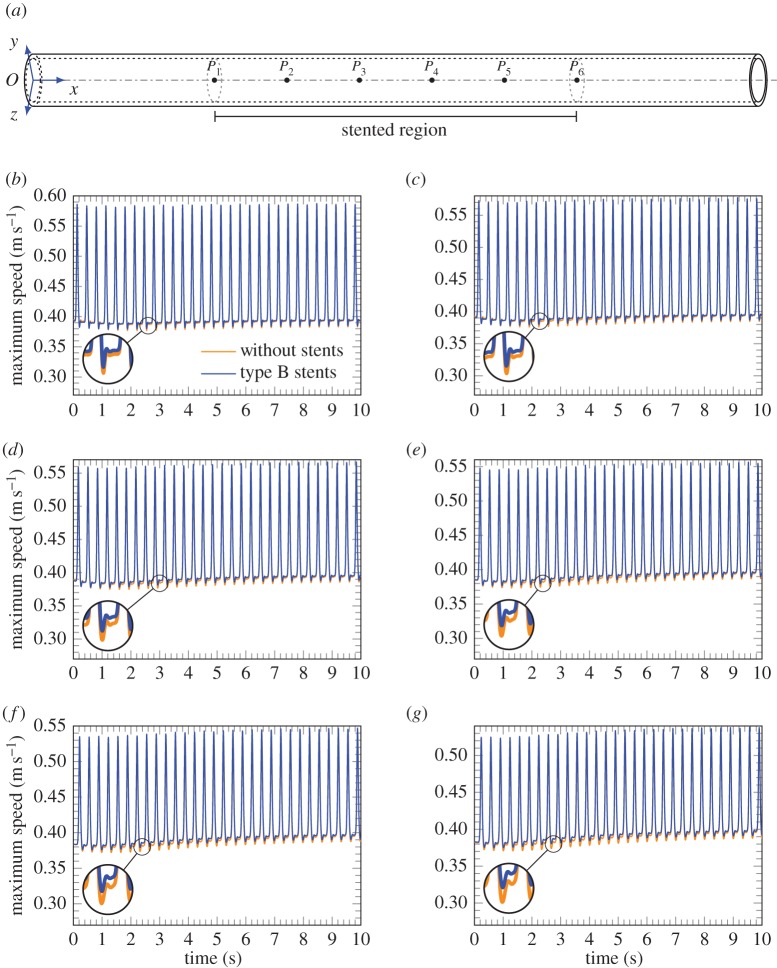
Evolution of the maximum fluid velocity at different points along the axis of the unstented artery and comparison with the case of a stented artery at 180 bpm, where the variation of amplitude Δ*A* is assumed equal to 0.5. (*a*) Scheme of the points in which the maximum fluid velocity is measured, (*b*) point *P*_1_ at *x*=25 mm, (*c*) point *P*_2_ at *x*=35 mm, (*d*) point *P*_3_ at *x*=45 mm, (*e*) point *P*_4_ at *x*=55 mm, (*f*) point *P*_5_ at *x*=65 mm and (*g*) point *P*_6_ at *x*=75 mm. (Online version in colour.)

Figures 13 and 14 illustrate the speed of the fluid flow on the axis of the cylindrical vessel as a function of time for different inlet frequencies. A comparison between unstented and stented blood vessels emphasizes the different transient response of these systems to a pulsating flow. The computations are presented for the case of type B stents.

It is observed that in the pass-band region the overall transient response of the stented artery does not show localization, and it converges to the time-harmonic mode slightly faster than in the case of the unstented softer system. It can be noted that for the case of the higher frequency shown in figure 13, the profile of the displacement curve varies in transition from the unstented to stented case. This profile clearly indicates the variation of the velocity of the flow due to the presence of stents in the system.

## 6. Conclusion

The paper has presented a novel approach to predictive dynamic modelling of a stented artery, which incorporates waveforms occurring due to the fluid–structure interaction. The outcomes of this study include description of regimes and deformations of blood vessels, which may have a detrimental effect on transmission of the blood flow.

The simplified, but mathematically advanced approach based on the concept of Bloch–Floquet waves, reveals several classes of dynamic deformations featured by a stented blood vessel. It has been demonstrated that both axisymmetric and non-axisymmetric deformations may be associated with so-called stop-bands, and therefore have a detrimental effect on the fluid flow through the stented vessel. The trapped modes are given special attention for clusters of stents separated by a finite distance, and asymptotic approximations are derived for predictive analysis of the associated waveforms.

The Bloch–Floquet wave theory has proved to be effective as the framework for time-harmonic modelling of waves in stented blood vessels. The approach proposed here enables one to evaluate qualitatively and quantitatively wave reflections from stents. In particular, it has been demonstrated that waves of certain frequencies can be blocked by stents placed in arteries. The geometry of stents has proved to be an important factor influencing the position of the first stop-band, which is critical for cardiovascular applications. In addition, the cross-linking of coils within the stent leads to the formation of additional stop-bands and may increase the stop-band width. Multiple stents separated by a finite distance further reduce the frequency at which the stop-bands can occur.

The transient analysis involving two-way fluid–structure interaction has been implemented for a stented blood vessel and a comparison with an unstented blood vessel is discussed in terms of blood flow. The greater understanding of the effects of stent design on the fluid–solid interaction will provide the researchers with more accurate modelling of this dynamic system. This will allow for further investigation into why certain arteries respond well to stenting, while others have difficulties.

## Supplementary Material

Waves and ﬂuid-solid interaction in stented blood vessels - Supplementary material

## Data accessibility

This paper contains no experimental data. All computational results are reproducible. Additional data on non-axisymmetric vibration modes and corresponding dispersion diagrams are included in the electronic supplementary material. Correspondence should be addressed to L.P.A, S.F. and A.B.M. (largani@liverpool.ac.uk, sara.frecentese@liverpool.ac.uk, abm@liverpool.ac.uk).
