# Supplementary material for: Waves and fluid–solid interaction in stented blood vessels
Source: Proc Math Phys Eng Sci. 2018 Jan 17;474(2209):20170670. doi: 10.1098/rspa.2017.0670 (PMC5806025; doi:10.1098/rspa.2017.0670)
Supplement: Waves and ﬂuid-solid interaction in stented blood vessels - Supplementary material [file rspa20170670supp1.pdf]

# Waves and fluid-solid interaction in stented blood vessels - Supplementary material

S. Frecentese[1], L.P. Argani[1], A.B. Movchan[1], N.V. Movchan[1], G. Carta[2], and M.L. Wall[3]

[1]*Department of Mathematical Sciences, University of Liverpool, Peach Street, Liverpool L69 7ZL, UK*
[2]*Department of Maritime and Mechanical Engineering, Liverpool John Moores University, 3 Byrom St., Liverpool L3 3AF, UK*
[3]*Russells Hall Hospital, The Dudley Group NHS Foundation Trust, Pensnett Road, Dudley DY1 2HQ, UK*

In the Supplementary material the dispersion curves for different types of stents are presented. In figures 1 and 2 the dispersion properties of axial and torsional modes are illustrated. In these cases it can be noted that no stop-bands appear.

In figures 3 and 4 the dispersion diagrams corresponding to modes involving trefoil and quatrefoiled flattening of the wall and to the flexural-torsional mode with rotation of the end sections are shown. Large stop-bands are observed for lower frequencies, hence it is unlikely that these modes occur within the standard pulsatile flow regime of human body.

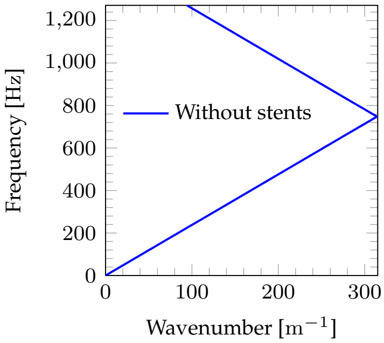


**(a)** *Unit cell without stents, torsional mode.*

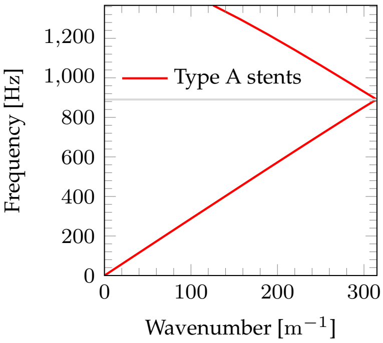


**(b)** *Unit cell with type A stents, torsional mode.*

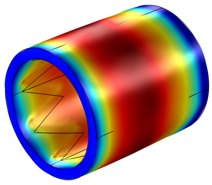

**(c)** *Representation of the torsional mode.*

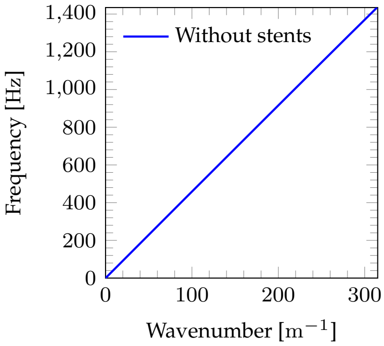


**(d)** *Unit cell without stents, axial mode.*

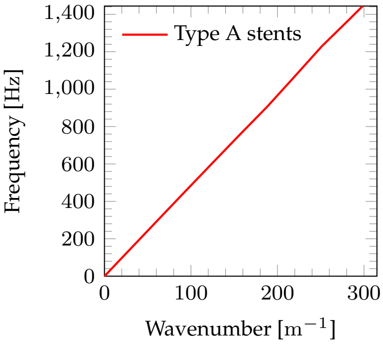


**(e)** *Unit cell with type A stents, axial mode.*

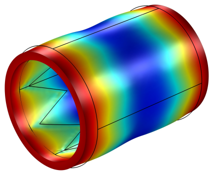

**(f)** *Representation of the axial mode.*

**Figure 1:** Dispersion curves for the torsional mode (a)-(c) and for the axial mode (d)-(f), for the unit cell without stents and with type A stents.

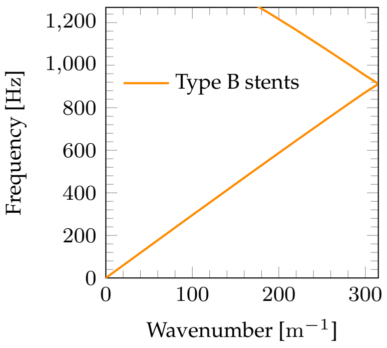


**(a)** *Unit cell with type B stents, torsional mode.*

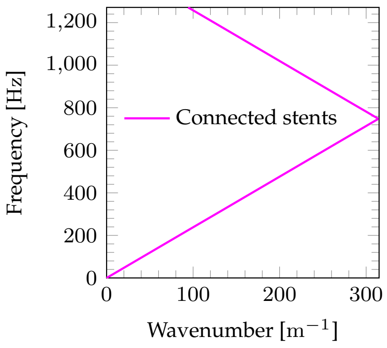


**(b)** *Unit cell with connected type A stents, torsional mode.*

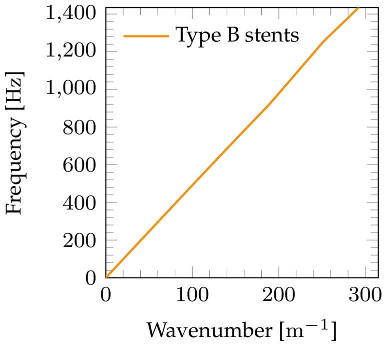


**(c)** *Unit cell with type B stents, axial mode.*

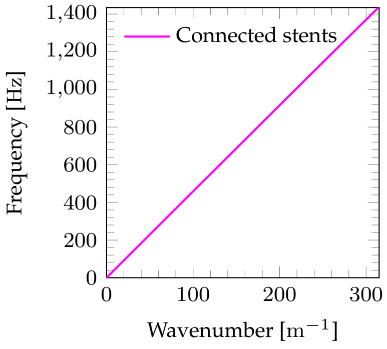


**(d)** *Unit cell with connected type A stents, axial mode.*

**Figure 2:** Comparison of the dispersion curves for the torsional mode (a)-(b) and for the axial mode (c)-(d), for the unit cell with different types of stents.

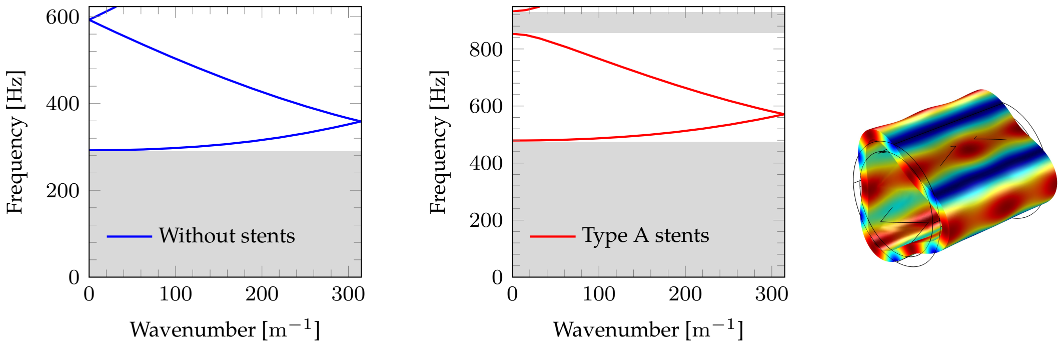


**(a)** *Unit cell without stents, mode involving trefoil flattening of the wall.*

**(b)** *Unit cell with type A stents, mode involving trefoil flattening of the wall.*

**(c)** *Representation of the mode involving trefoil flattening of the wall.*

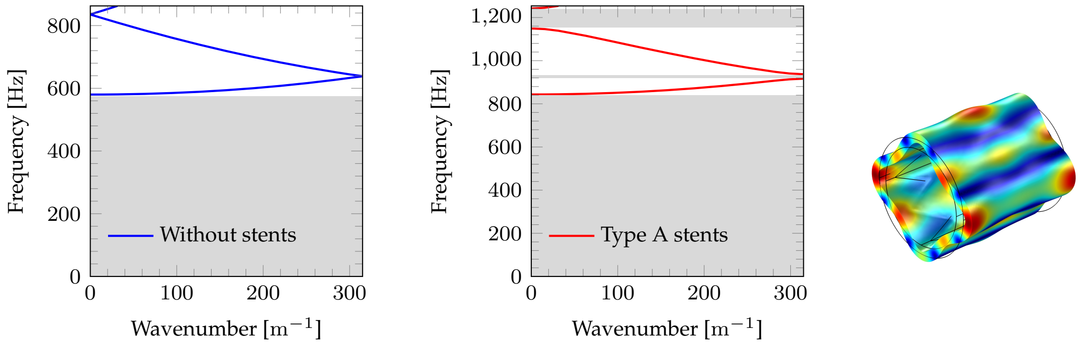


**(d)** *Unit cell without stents, mode involving quatrefoiled flattening of the wall.*

**(e)** *Unit cell with type A stents, mode involving quatrefoiled flattening of the wall.*

**(f)** *Representation of the mode involving quatrefoiled flattening of the wall.*

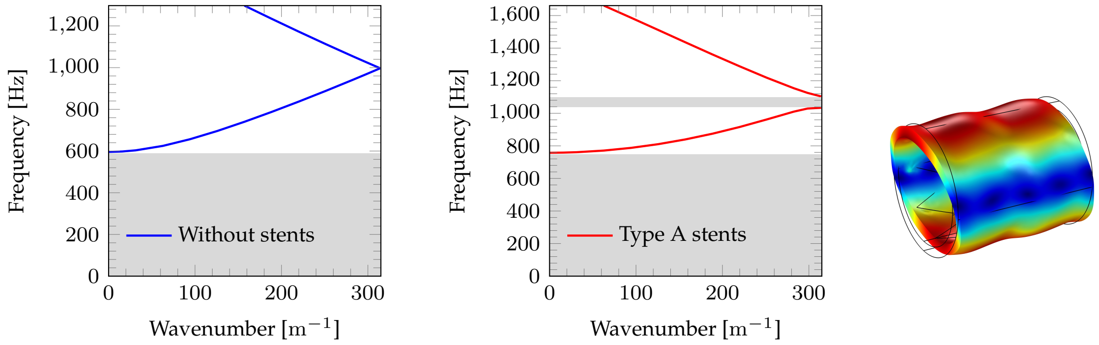


**(g)** *Unit cell without stents, flexural-torsional mode with rotation of the end sections.*

**(h)** *Unit cell with type A stents, flexural-torsional mode with rotation of the end sections.*

**(i)** *Representation of the flexural-torsional mode with rotation of the end sections.*

**Figure 3:** Dispersion curves for the modes involving trefoil (a)-(c) and quatrefoiled (d)-(f) flattening of the wall and for the flexural-torsional modes with rotation of the end sections (g)-(i), for the unit cell without stents and with type A stents. The shaded zones denote the stop-bands.

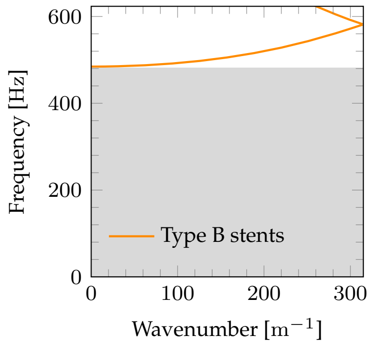


**(a)** *Unit cell with type B stents, mode involving trefoil flattening of the wall.*

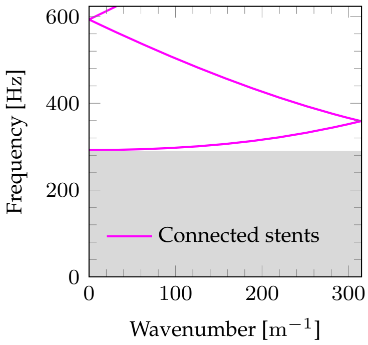


**(b)** *Unit cell with connected type A stents, mode involving trefoil flattening of the wall.*

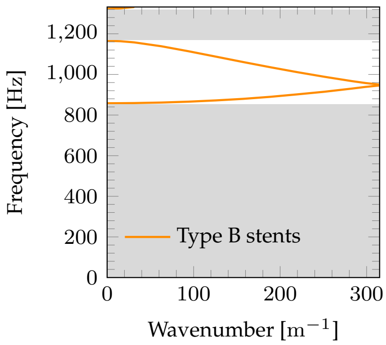


**(c)** *Unit cell with type B stents, mode involving quatrefoiled flattening of the wall.*

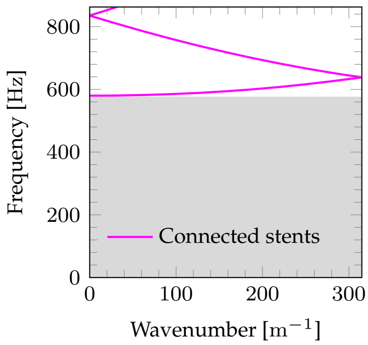


**(d)** *Unit cell with connected type A stents, mode involving quatrefoiled flattening of the wall.*

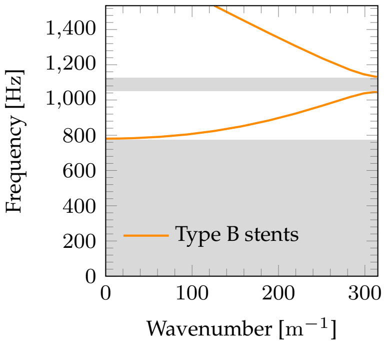


**(e)** *Unit cell with type B stents, flexural-torsional mode with rotation of the end sections.*

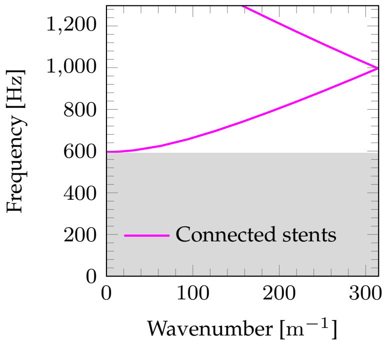


**(f)** *Unit cell with connected type A stents, flexural-torsional mode with rotation of the end sections.*

**Figure 4:** Comparison of the dispersion curves for the modes involving trefoil (a)-(b) and quatrefoiled (c)-(d) and for the flexural-torsional modes with rotation of the end sections (e)-(f), for the unit cell with different types of stents. The shaded zones denote the stop-bands.
